# Shikonin Kills Glioma Cells through Necroptosis Mediated by RIP-1

**DOI:** 10.1371/journal.pone.0066326

**Published:** 2013-06-28

**Authors:** Chuanjiang Huang, Yinan Luo, Jingwei Zhao, Fuwei Yang, Hongwei Zhao, Wenhai Fan, Pengfei Ge

**Affiliations:** 1 Department of Neurosurgery, First Bethune hospital of Jilin University, Changchun, China; 2 Department of Neurosurgery, China-Japan Union hospital of Jilin University, Changchun, China; 3 Department of Neurosurgery, Zhongshan hospital affiliated to Dlian University, Dalian, China; University of Pittsburgh, United States of America

## Abstract

**Background and Purpose:**

Shikonin was reported to induce necroptosis in leukemia cells, but apoptosis in glioma cell lines. Thus, it is needed to clarify whether shikonin could cause necroptosis in glioma cells and investigate its underlying mechanisms.

**Methods:**

Shikonin and rat C6 glioma cell line and Human U87 glioma cell line were used in this study. The cellular viability was assayed by MTT. Flow cytometry with annexin V-FITC and PI double staining was used to analyze cellular death modes. Morphological alterations in C6 glioma cells treated with shikoinin were evaluated by electronic transmission microscopy and fluorescence microscopy with Hoechst 33342 and PI double staining. The level of reactive oxygen species was assessed by using redox-sensitive dye DCFH-DA. The expressional level of necroptosis associated protein RIP-1 was analyzed by western blotting.

**Results:**

Shikonin induced cell death in C6 and U87 glioma cells in a dose and time dependent manner. The cell death in C6 and U87 glioma cells could be inhibited by necroptosis inhibitor necrotatin-1, not by pan-caspase inhibitor z-VAD-fmk. Shikonin treated C6 glioma cells presented electron-lucent cytoplasm, loss of plasma membrane integrity and intact nuclear membrane in morphology. The increased ROS level caused by shikonin was attenuated by necrostatin-1 and blocking ROS by anti-oxidant NAC rescued shikonin-induced cell death in both C6 and U87 glioma cells. Moreover, the expressional level of RIP-1 was up-regulated by shikonin in a dose and time dependent manner as well, but NAC suppressed RIP-1 expression.

**Conclusions:**

We demonstrated that the cell death caused by shikonin in C6 and U87 glioma cells was mainly via necroptosis. Moreover, not only RIP-1 pathway, but also oxidative stress participated in the activation of shikonin induced necroptosis.

## Introduction

Malignant gliomas account for approximately 70% of the 22,500 new cases of malignant primary brain tumors that are diagnosed in adults in the United States each year [1]. Although relatively uncommon, malignant gliomas are associated with high morbidity [2]. It is very difficult to eliminate malignant glioma cells, because surgical operation can not remove them out radically and they are resistant to postoperative radiotherapy and chemotherapy. Recent studies show that resistance to apoptosis is the major factor that makes malignant glioma cells survive current clinically used medicines or radiotherapy [3]. Thus, it is needed to find new medicines that could induce glioma cell death not via apoptosis pathway [4].

Currently, necroptosis (a type of programmed necrosis) is found to be a new form of programmed cell death that is different with apoptosis [5]. In morphology, necroptosis has the characteristics resembling to unregulated necrosis including loss of plasma membrane integrity, gain in cell volume and swelling organelles [6]. However, necroptosis exhibits a signaling pathway that requires the involvement of receptor interaction protein kinases and can be specifically inhibited by necrostatin-1 [7]. Recently, necroptosis has been found to be involved in some pathological conditions. It not only contributes to ischemic injury in brain, heart and kidney [8]–[10], but also accelerates cancer cell death or enhances the sensitivity of tumor cells to anti-cancer treatment [11]–[13]. Particularly, necroptosis is able to overcome resistance to cancer drugs mediated by P-glycoprotein, Bcl-2, and Bcl-xL in cancer cell lines [14]. Thus, necroptosis has become a new target to induce tumor cell death.

Shikonin is a naphthoquinone isolated from Lithospermum erythrorhizon, and has been broadly used for thousands of years in traditional Chinese medicine for the treatment of burns, carbuncles, measles, macular eruptions, and sore throats [15]. Accumulating evidences have demonstrated that shikonin could induce apoptosis in various types of tumor cell lines such as breast cancer, hepatocellular carcinoma and osteosarcoma [15]–[17]. Particularly, it was reported recently that glioma cell death caused by shikonin is also via apoptosis pathway [18]. However, shikonin has been found to cause necroptosis in leukemia cell lines [14]. Thus, whether shikonin could induce necroptosis in glioma cells is still needed to be examined as well. Clarifying this issue would help us to understand the mechanism underlying the anti-glioma effects of shikonin. Therefore, in this study, we use rat C6 glioma cells and Human U87 glioma cells to investigate this issue.

## Materials and Methods

### Reagents

Shikonin and Nec-1(necrostatin-1) were both from Sigma (St. Louis, MO, USA). Shikonin was dissolved in PBS to a storage concentration of 50 μmol/L, and Nec-1 was dissolved in PBS to a storage concentration of 1 mmol/L. DMEM medium was from Gibco (Rockville, MD, USA). Fetal bovine serum (FBS) from Life Technologies (Grand Island, NY, USA). Protein concentration assay kit from Bio-rad Laboratory (Hercules, CA, USA). ECL Western blotting detection reagents from Amersham Company (Piscataway, NJ, USA). PVDF membranes from Millipore Company (Billerica, MA, USA). Other reagents were from Sigma Company (St. Louis, MO, USA).

### Cell line and culture

Rat C6 glioma cells and Human U87 glioma cells were obtained from Shanghai Institute of Cell Biology, Chinese Academy of Sciences (Shanghai, China). Cells were cultured in DMEM supplemented with 10% fetal bovine serum, 2 mmol/L glutamine (Gibco, Grand Island, NY, USA), penicillin (100 U/mL) and streptomycin (100 μg/mL), and maintained at 37°C and 5% CO2 in a humid environment. Cells in the mid-log phase were used in the experiments.

### Cell viability assay

C6 (3×10^5^ cells/well) and U87 (1.5×10^5^ cells/well) glioma cells were seeded onto 96-well microplate and cultured 24 h. PBS was added into the control group and shikonin was added into experimental group to reach the final concentration. Cellular viability was assessed using an MTT assay after shikonin treatment at indicated time point. The absorbance value (*A*) at 570 nm was read using an automatic multi-well spectrophotometer (Bio-Rad, Richmond, CA, USA).

Two groups of glioma cells from the same cell line were treated with shikonin at lower or higher concentration, respectively; other two groups of glioma cells were treated 1 h with 100 µmol/L nec-1 or 40 µmol/L z-VAD-fmk prior to co-incubation with shikonin at indicated concentration. Additionally, another two groups of glioma cells were treated only with 100 µmol/L nec-1 or 40 µmol/L z-VAD-fmk at corresponding time point.

### Hoechst 33342 and PI (propidium iodide) staining

C6 glioma cells (3×10^5^ cells/well) were allowed to grow on coverslips in 6-well culture plates for 24 h. The cells were then treated with either PBS (control) or shikonin at indicated concentration for 3 h at 37°C. Cells growing on glass coverslips were fixed in methanol for 5 min at room temperature. The fixed cells were washed twice with PBS and then incubated with Hoechst 33342 (1 μg/mL) for 5 min at room temperature, following incubation with PI (5 μg/mL) for 15 min at room temperature and observed under fluorescence microscope. After a final wash in PBS, samples were visualized with the aid of EPI fluorescence microscopy at 200× magnification (Nikon eclipse E800). Excitation wavelength of 330–380 nm was applied for Hoechst dye and 450–490 nm for PI. Apoptotic cells were identified on the basis of morphologic changes in their nuclear assembly by observing chromatin condensation and fragment staining by the Hoechst dye. Necroptotic cells were identified based on the positive staining with PI and without apoptotic nuclear morphology with Hoechst dye. In each case at least four microscopic fields were photographed randomly. The experiments were repeated at least three times.

### Transmission electron microscopy

C6 glioma cells were cultured and treated with shikonin at indicated concentration and harvested using 0.25% trypsin, washed with PBS. Then the cells were collected by centrifugation for 10 minutes at 2000 rev/min and treated as described by Ge et al [19]. Briefly, the cells were fixed in ice-cold 2.5% glutaraldehyde in PBS (pH 7.3), rinsed with PBS and post-fixed in 1% osmium tetroxide with 0.1% potassium ferricyanide, dehydrated through a graded series of ethanol (30–90%) and embedded in Epon (Energy Beam Sciences, Agawam, MA, USA). Semithin (300 nm) sections were cut using a Reichart Ultracut, stained with 0.5%toluidine blue and examined under a light microscope. Ultrathin sections (65 nm) were stained with 1% uranyl acetate and 0.1% lead citrate, and examined on a JEM2000EX transmission electron microscope (JEOL, Pleasanton, CA, USA).

### Measurement of intracellular ROS levels

The average level of intracellular ROS in C6 and U87 glioma cells was evaluated in cells loaded with the redox-sensitive dye DCFH-DA (Molecular Probes, Eugene, OR). It was examined at 3 h for the cells treated with shikonin alone, or for the cells incubated with shikonin following pretreatment with 100 µmol/L Nec-1 or 400 µmol/L NAC. The cells treated with PBS were used as control. All the experimental cells were washed twice in a phosphate-buffered saline (PBS) and stained in the dark for 30 min with 20 μmol/L DCFH-DA and harvested. Cells were dissolved with 1% Triton X-100, and fluorescence was measured at an excitation wavelength of 485 nm and an emission wavelength 530 nm using a fluorescence spectrometer (HTS 7000, Perkin Elmer, Boston, MA). The ROS levels were expressed as arbitrary unit/mg protein, then as the percentage of control.

### Detection of cell death by flow cytometry

After 12 h of starvation in serum free DMEM/F12, except for the control group, C6 glioma cells were treated with 3 µmol/L and 6 µmol/L shikonin and U87 cells were treated with 5 µmol/L and 10 µmol/L shikonin. The other groups of C6 or U87 glioma cells were treated 1 h with 100 µmol/L nec-1, 400 µmol/L NAC or 40 µmol/L z-VAD-fmk prior to co-incubation with shikonin, respectively.

Cellular death was evaluated through annexin V-FITC apoptosis detection kit (Invitrogen, Grand Island, NY) as described by the manufacture's instruction. Briefly, glioma cells were collected 3 h after the target compounds treatment, and washed twice with PBS, then resuspended in 400 μL 1× binding buffer (10 mM HEPES/NaOH, 140 mM NaCl, 2.5 mM CaCl_2_, pH 7.4). Cells (100 μL) were transferred to a 5-mL culture tube containing 5 μL of annexin V-FITC and 10 μL of propidium iodide, and then incubated for 15 min at room temperature in the dark. After 1× binding buffer was added into each tube, the stained cells were analyzed by flow cytometry (FACScan, Bection Dickinson, San Jose, CA). The rate of cell death was analyzed using CELLquest software (Bection Dickinson). Data acquisition was conducted by collecting 20,000 cells per tube and the numbers of viable and dead cells were determined for each experimental condition.

### Gel Electrophoresis and Western Blotting

C6 and U87 glioma cells were cultured, harvested and washed as in the section of detection of cell death by flow cytometry. After centrifugation 10 min at 1,000g, the cell pellets were suspended in ice-cold buffer containing 15 mmol/L Tris, pH 7.6, 250 mmol/L sucrose, 1 mmol/LMgCl_2_, 2.5 mmol/L EDTA, 1 mmol/L EGTA (ethylene glycol-bis (b-amino ethylether) tetraacetic acid), 1 mmol/L dithiothreitol, 1.25 mg/mL pepstatin A, 10 mg/mL leupeptin, 2.5 mg/mL aprotinin, 1.0 mmol/L phenylmethylsulfonyl fluoride (PMSF), 0.1 mmol/L Na_3_VO_4_, 50 mmol/L NaF, and 2 mmol/L Na_4_P_2_O_7_ and homogenated with a glass Pyrex microhomogenizer (20 strokes). Homogenates were centrifuged at 10,000 g at 4°C for 10 min to obtain supernatant. The protein content of the supernatant was determined using Bio-Rad protein assay kit.

Equal protein amounts were electrophoresed on 10% sodium dodecyl sulfate-polyacrylamide gels and then transferred to PVDF membranes. The membranes were blocked with 3% bovine serum albumin in TBS for 30 min and then incubated overnight at 4°C with anti-RIP-1 antibody (1∶5000) and β-actin (1∶3000) (Abcam, Cambridge, MA). After being incubated with horseradish peroxidase-conjugated goat anti-rabbit IgG (1∶2000), blots were washed and immunoreactive proteins were visualized on a Kodak X-omat LS film (Eastman Kodak Company, New Haven CT) with an enhanced chemiluminescence (Amersham Biosciences, Piscataway NJ). Densitometry was performed with Kodak ID image analyses software (Eastman Kodak Company).

### Statistical analysis

All data represent at least 4 independent experiments and are expressed as mean±SD. Statistical comparisons were made using ANOVA. *P*-values of less than 0.05 were considered to represent statistical significance.

## Results

### Shikonin inhibited cellular viability in glioma cells

**Figure 1:**
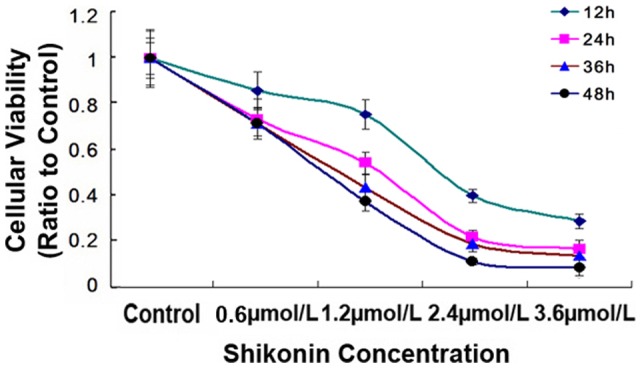
Long-term effects of shikonin on the cellular viability of C6 glioma cells. MTT assay showed that the decreased cellular viability of C6 glioma cells caused by shikonin was dependent on shikonin concentration and incubation time. *: *p*<0.01 versus control group.

In order to investigate the effects of shikonin on glioma cells, MTT was used to assay the alterations of cellular viability. As shown in figure 1, the viability of C6 glioma cells decreased with extension of incubation time with shikonin and elevation of shikonin concentration.

**Figure 2:**
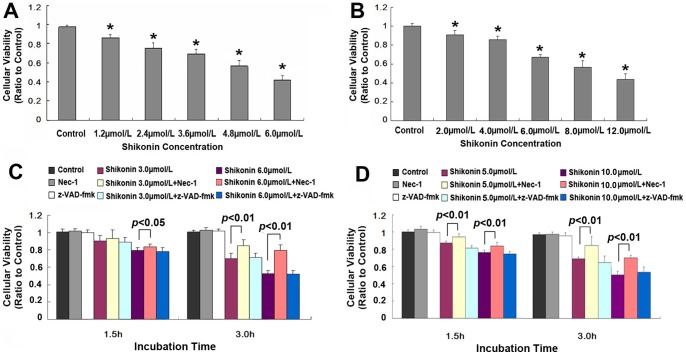
Short-term effects of shikonin on the cellular viability of C6 and U87 glioma cells. MTT assay showed that the viability of C6 (A) and U87 (B) glioma cells decreased in a dose dependent manner when incubated 3 hours with shikonin. However, the inhibitory effects of shikonin on the viability of C6 (C) and U87 (D) glioma cells was suppressed significantly by pretreatment with Nec-1, but not by z-VAD-fmk.

However, under light microscope, C6 glioma cells were found to present the morphological features of dying cells (data not shown), we thus limited the incubation time of C6 glioma cells with shikonin to 3 hours. As shown in Figure 2A, after 3 hours incubation with shikonin at the concentration of 1.2 µmol/ L, 2.4 µmol/ L, 3.6 µmol/L, 4.8 µmol/L and 6.0 µmol/L, the average viability of C6 cells reduced to 88.2%, 77.5%, 71.1%, 57.6 and 44.4% when compared with that in control group. On the basis of above data, the IC50 value of shikonin on C6 cells was 5.85 µmol/L, and 6.0 µmol/L was used as the IC50 value in the subsequent study. In order to examine further the inhibitory effects of shikonin on the viability of glioma cells, we used U87 glioma cells to incubate with shikonin. As figure 2B showed, after 3 hours incubation, the average viability of U87 cells reduced to 91.2%, 84.6%, 67.1%, 57.2 and 43.4% at the shikonin concentration of 2.0 µmol/ L, 4.0 µmol/ L, 6.0 µmol/L, 8.0 µmol/L and 12.0 µmol/L, respectively. Thus, the IC50 value of shikonin on U87 cells was about 10.0 µmol/L and was used as the IC50 value in the following studies. These results indicated that shikonin inhibited the viability of C6 cells in a dose dependent manner.

Additionally, as shown in Fig 2C, when incubation time was extended form 1.5 h to 3.0 h, the viability of C6 cells decreased from 89.7% to 68.2% at 3.0 µmol/L shikonin, and from 78.6% to 52.2% at 6.0 µmol/L shikonin. Similarly, the viability of U87 cells decreased from 87.7% to 75.6% at 5.0 µmol/L shikonin, and from 68.7% to 50.9% at 10.0 µmol/L shikonin (Fig 2D). This indicated that the inhibitory effect of shikonin on glioma cells was dependent on incubation time as well.

### Shikonin induced glioma cell death was attenuated by Nec-1

As figure 2 C showed, the decreased viability of C6 glioma cells caused by 3.0 µmol/L and 6.0 µmol/L shikonin was improved by pretreatment with Nec-1 (a specific inhibitor of necroptosis) to 92.3% and 82.9% at 1.5 h and 84.4% and 78.6% at 3.0 h, respectively. Similarly, the viability of U87 glioma cells was elevated by Nec-1 to 91.6% and 81.5% at 1.5 h, and 81.8% and 71.2% at 3.0 h, respectively (Fig 2D). By contrast, z-VAD-fmk (an inhibitor of caspase-dependent apoptosis) did not affect the viability of C6 and U87 glioma cells treated with shikonin either at higher or lower concentration (Fig 2 C and D). This indicated that shikonin induced cell death in glioma cells might be via necroptosis pathway.

**Figure 3:**
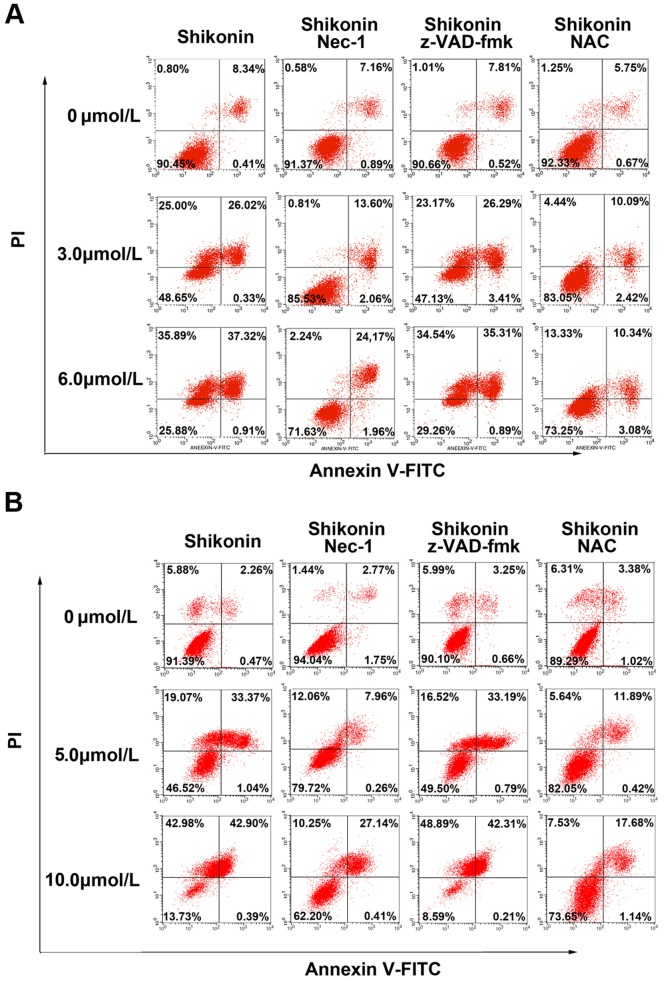
Flow cytometry analysis of glioma cell death mode caused by shikonin. (A) Representative image of flow cytometry of C6 (A) and U87 glioma cells (B). After 3 hours incubation either with lower or higher concentration of shikonin, both the percentage of necrotic cells and late apoptotic cells increased significantly. The necrotic and late apoptotic cells were significantly inhibited by pretreatment with Nec-1, but did not suppress by pre-incubation with z-VAD-fmk. These data indicated that Nec-1 blocked shikonin induced necrosis in glioma cells. Additionally, late apoptotic cells did not reduce when they were pretreated with z-VAC-fmk but attenuated by Nec-1, indicating they were necrotic cells as well. Further, the necroptotic cells were suppressed by antioxidant NAC.

Further, flow cytometry with Annexin V and PI double staining was performed to investigate the death modes caused by shikonin in glioma cells. As figure 3A showed, after 3 hours incubation with 3.0 µmol/L and 6.0 µmol/L shikonin, the necrosis (Annexin V−/Pl+) rates of C6 glioma cells were 25.0% and 35.89%, and late apoptosis (Annexin V+/Pl+) rates were 26.02% and 37.32%, respectively. However, no significant alteration was found in the cells at early apoptosis stage (Annexin V+/Pl−). This suggested that necrotic C6 cells increased with elevation of shikonin concentration. By contrast, pretreatment with Nec-1 made the necrotic cells caused by 3.0 µmol/L and 6.0 µmol/L shikonin reduce to 0.81% and 2.24%, respectively. This suggested that Nec-1 effectively blocked shikonin induced necrosis. Similarly, the cell at late apoptosis stage was also attenuated by Nec-1 to 13.6% and 24.17%, but not by z-VAD-fmk, indicating part of the cells at the stage of late apoptosis might be necrotic cells. Moreover, similar findings were revealed in U87 glioma cells (Fig 3B). Thus, these results indicated that the death mode of glioma cells caused by shikonin is necroptosis, not apoptosis.

### Shikonin induced necrosis-like morphological alterations in C6 glioma cells

**Figure 4:**
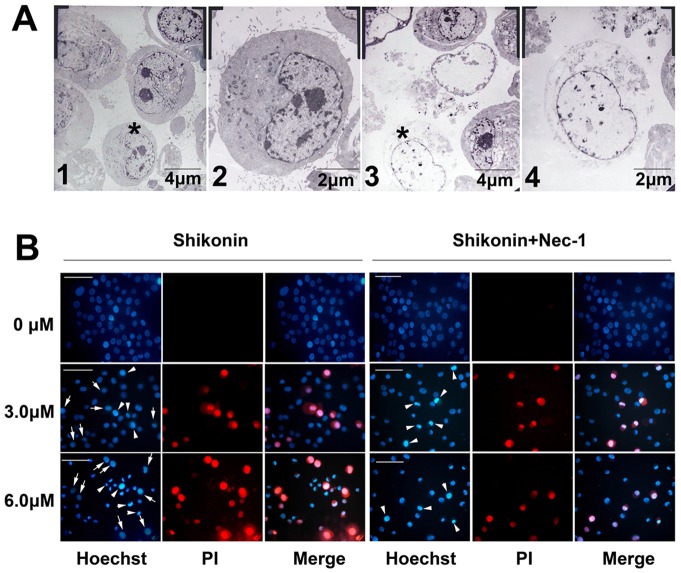
Morphological changes in C6 glioma cells induced by shikonin. (A) Images under transmission electron microscope. A1, normal C6 glioma cells; A2, enlarged cellular image labeled with asterisk in A1; A3, C6 cells treated 3h with 6.0 μmol/L shikonin; A4, enlarged cellular image labeled with asterisk in A3. Compared with normal C6 glioma cells, morphological features of necrosis such as electron-lucent cytoplasm, swollen or disrupted organelles, intact nuclear membrane and loss of plasma membrane were found in the C6 cells treated with shikonin. (B) Nuclear images under fluorescence microscope. Blue color represented being stained with Hoechst 33342, and red color represented PI staining. The nucleus in C6 glioma cell treated with 0 µmol/L shikonin or Nec-1 alone showed round figure and homogenous blue color. In the shikonin treated groups, part of the nuclei stained with red color showed no changes in the nuclear figure or size and density of blue color, indicating they were necrotic cells (arrow). By contrast, another part of the nuclei with red staining displayed increased fluo-density in blue color and condensed nuclear size, indicating that they were apoptotic cells (arrow head). Pretreatment with Nec-1 decreased necrotic cells, but apoptotic cells still could be found. Scale bar  = 20 µm.

For clarifying the death mode in C6 glioma cells treated with shikonin, we performed morphological investigation. Under transmission electron microscope, normal C6 glioma cells displayed microvilli protruding from the entire surface, a smoothly outlined nucleus with chromatin in the form of heterochromatin and well-preserved cytoplasmic organelles. By contrast, shikonin treated C6 glioma cells presented electron-lucent cytoplasm, loss of plasma membrane integrity, and intact nuclear membrane (Fig 4A), which were consistent with the characteristics of necrosis [20]. This indicated that shikonin treated C6 glioma cells showed alterations similar to necrosis in morphaology.

Further, necrosis was confirmed by using a combination of Hoechst 33342/PI double staining to determine the nuclear morphology and membrane integrity. Hoechst 33342, as bisbenzimidazole dye, is known to penetrate the plasma membrane and stain DNA in cells without permeabilization, and a stronger blue fluorescence can be observed in apoptotic cells compared with non-apoptotic cells [21]. Necrotic cells can be detected by the uptake of PI which indicates damage of cell membrane, while apoptotic cells and normal cells are lack of PI uptake due to the integrity of cell membrane. Nuclear condensation or fragmentation along with PI uptake can be observed in late apoptotic cells that sustained some terminal damage to the cell membrane before they were cleared [22]. Thus, Hoechst 33342 stains all nuclei and PI stains nuclei of cells with a disrupted plasma membrane. Compared with normal C6 glioma cells showing round nuclei with dark blue color, part of the C6 cells treated by shikonin had nuclei in normal size but stained with dark blue and red color (pointed by arrow in figure 4B), indicating they were necrotic cells. Another part of shikonin treated C6 cells presented shrunken nuclei in light blue color, with being stained with red color, indicating they were apoptotic cells (pointed by arrowhead in figure 4B). However, pretreatment with necroptosis inhibitor Nec-1 made significant reduction in the cells with nuclei in normal size and being stained with dark blue and red color, but the cells in light blue and red color remained. Thus, based on these morphologic findings, we thought the cell death caused by shikonin might be associated with necroptosis. These data indicated as well that Nec-1 suppressed cells in necroptosis but not in apoptosis.

### Inhibition of oxidative stress suppressed shikonin induced necroptosis

**Figure 5:**
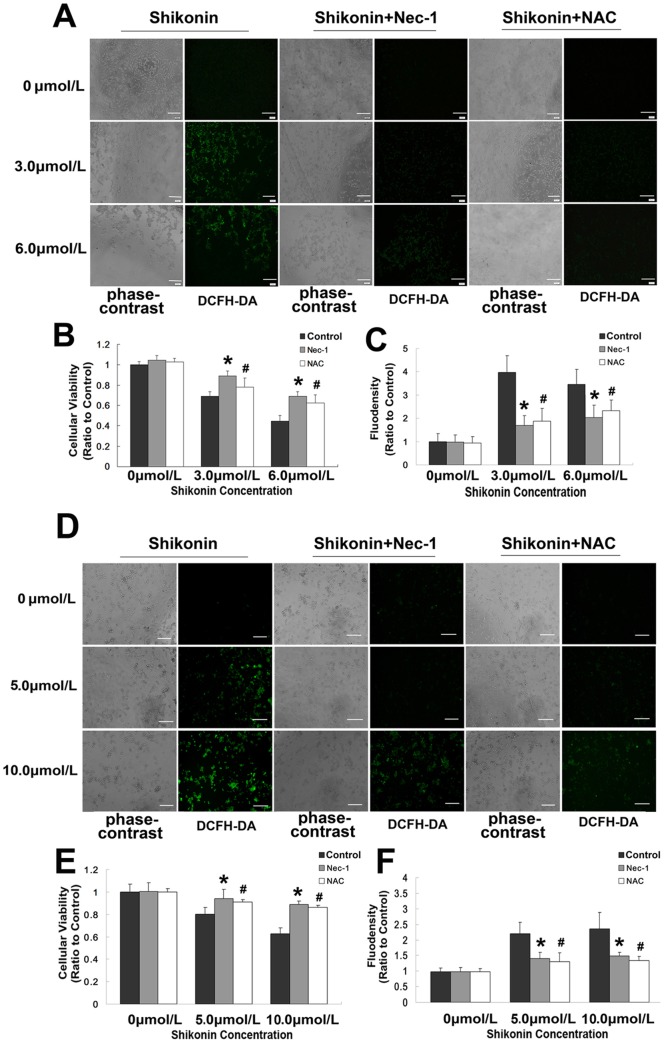
Changes in ROS production and cellular viability in glioma cells caused by shikonin. The left panels in A and D were the images of C6 and U87 glioma cells under phase contrast microscope, and the right panel were the fluorescence microscopic images of ROS generation by shikonin using DCFH-DA staining (4X). B and E were statistical analysis of cellular viability in C6 and U87 glioma cells. C and F were statistical analysis of fluodensity. The images showed that the ROS level increased significantly in both C6 and U87 glioma cells treated with shikonin while cellular viability decreased when compared with control group. However, no significant difference in fluodensity was found either in C6 or U87 cells between lower or higher concentration of shikonin groups. By contrast, pretreatment with Nec-1 or NAC inhibited the production of ROS and rescued C6 and U87 glioma cell death caused by shikonin. Scale bar  = 40 μm. *: *p*<0.01 versus control group; #: *p*<0.01 versus control group.

It was previously reported that RIP-1 could modulate oxidative stress [23]. We thus examined the shikonin induced changes in the level of reactive oxygen species (ROS) in glioma cells to investigate the influence of oxidative stress on necroptosis. As shown in figure 5A and C, compared with that in control group, the ROS level increased to 3.97±0.71 times and 3.36±0.46 times when C6 glioma cells incubated 3 h with 3.0µmol/L and 6.0µmol/L shikonin, respectively. By contrast, the elevated ROS level was inhibited effectively not only by 100 µmol/L Nec-1, but also by 400 µmol/L antioxidant NAC (Figure 5 A and C). Meanwhile, MTT assay showed that cellular death caused by shikonin in C6 glioma cells was significantly suppressed by Nec-1 and NAC (Figure 5 A and B). Similarly, as shown in figure 3A, flow cytometry demonstrated that pre-treatment with NAC made the necrotic cells decrease from to 25.0% to 4.44% at 3.0 µmol/L shikonin, and from 35.89% to13.33% at 6.0 µmol/L shikonin. Moreover, similar findings were revealed in U87 glioma cells (Fig 5 D, E and F, and Fig 3B). Thus, these data indicated that shikonin induced necroptosis in glioma cells was associated as well with oxidative stress.

### Shikonin up-regulated the expression of necroptosis associated protein RIP-1

**Figure 6:**
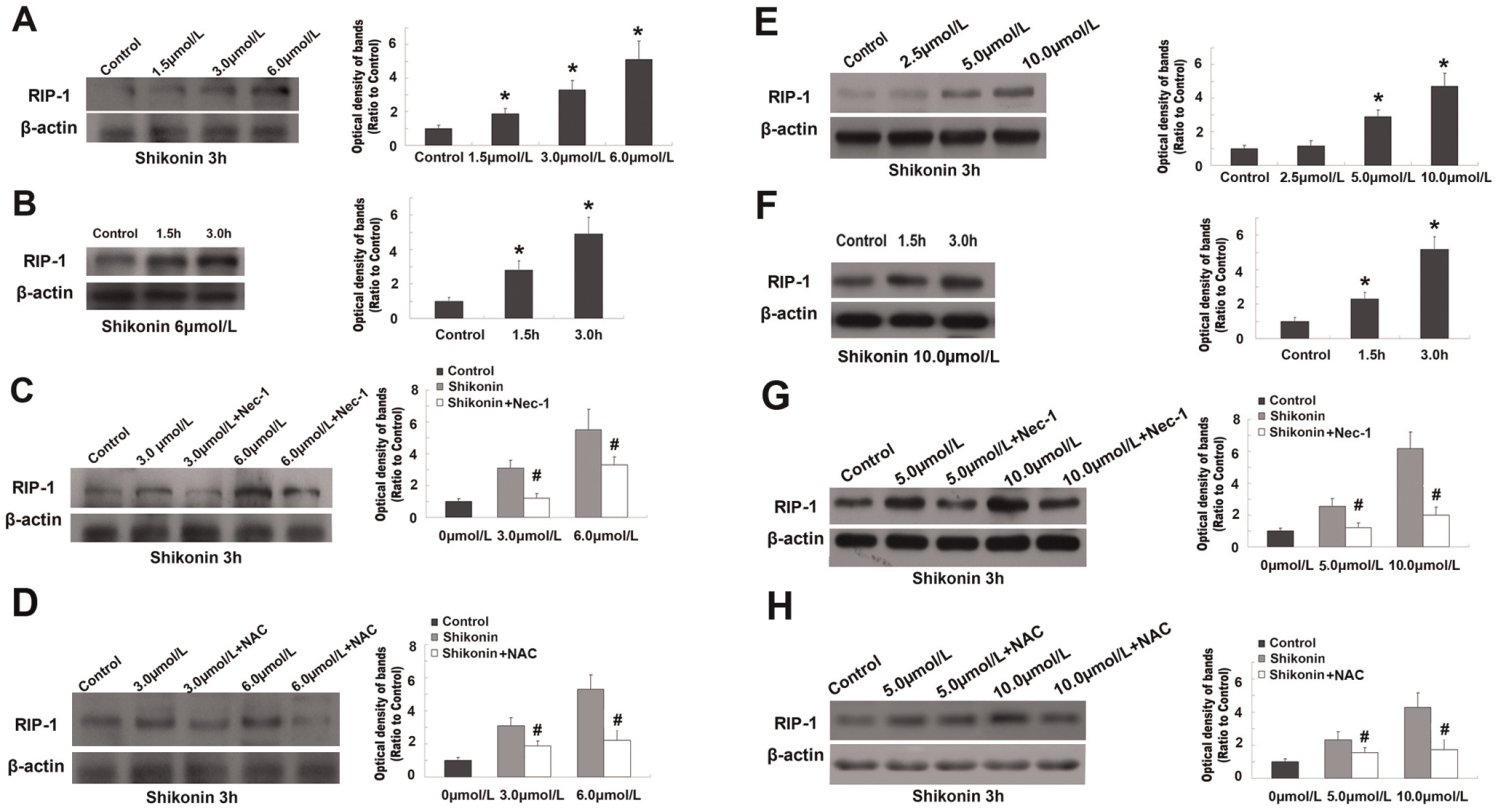
Western blotting analysis of the expression of RIP-1. The expressional level of RIP-1 increased significantly in C6 (A) and U87 (E) glioma cells when shikonin concentration was elevated. The expression of RIP-1 was up-regulated markedly when the incubation of C6 (B) or U87 (F) glioma cells with shikonin was elongated from 1.5 h to 3.0h. Pretreatment with Nec-1 attenuated the increased expression of RIP-1 caused by shikonin either at lower or higher concentration in C6 (C) and U87 (G) glioma cells. Pretreatment with NAC made the increased level of RIP-1 decrease in both C6 (D) and U87 (H) glioma cells when compared with shikonin group. *: *p*<0.01 versus control group; #: *p*<0.01 versus shikonin treated group.

For studying the molecular mechanism underlying the inhibitory effects of shikonin on glioma cell viability, we examined the expressional level of RIP-1 that was thought to play a crucial role in initiating necroptosis [24]. We found that, the expressional level of RIP-1 was up-regulated significantly in C6 and U87 glioma cells when shikonin concentration increased (Fig 6 A and E), and incubation time extended from 1.5 h to 3.0 h (Fig 6 B and F). By contrast, pretreatment with Nec-1 suppressed the expression of RIP-1 caused by shikonin. At 3 h incubation time, the expressional levels of RIP-1 in C6 glioma cells induced by 3.0 µmol/L and 6.0 µmol/L shikonin were 3.12±0.39 times and 5.49±1.25 times as high as that in the control group, respectively. However, they became 1.22±0.28 times and 3.32±0.47 times when pretreated by Nec-1(Fig 6 C). Similarly, the RIP-1 expression in U87 glioma cells was up-regulated significantly by 5.0 µmol/L and 10.0 µmol/L shikonin, and suppressed by pretreatment with Nec-1(Fig 6 G). These results indicated that shikonin caused glioma cell death was via up-regulating the expression of RIP-1, and Nec-1 rescued cell death caused by shikonin via inhibition of RIP-1 expression.

For clarifying the relationship between antioxidant NAC and RIP-1, we performed western blotting to examine the effects of NAC on the expression of RIP-1. We found that, pretreatment with 400 µmol/L NAC made the increased level of RIP-1 decrease markedly either in C6 or U87 glioma cells when compared with control group (Fig 6 D and H). This indicated that the protection of NAC on glioma cell death caused by shikonin is also associated with down-regulation of RIP-1.

## Discussion

In this study, we found that cell death and expressional level of RIP-1 caused by shikonin in C6 or U87 glioma cells were both dependent on the concentration of shikonin and the incubation time with shikonin. Shikonin treated C6 glioma cells presented necrosis-like characteristics in morphology. Necroptosis inhibitor Nec-1 inhibited cellular death in C6 and U87 glioma cells via attenuating the increased expressional level of RIP-1 and suppressing the elevated production of ROS induced by shikonin. Additionally, antioxidant NAC rescued shikonin induced glioma cell death. Our results indicated that shikonin caused necroptosis in glioma cells was associated with the up-regulated expression of RIP-1 and oxidative stress.

It has been established that necroptosis is a new form of programmed cell death, and some chemicals such as D-galactose, cyclosporin A, staurosporine and palmitic acid has been demonstrated to induce cell death via activation of necroptosis [13], [25]–[27]. Morphological alteration is an important criterion for evaluating cellular death mode. Under transmission electron microscope, apoptosis and necroptosis have totally different features. Apoptosis often presented pyknosis, chromatin condensation, nuclear fragmentation, and the appearance of apoptotic bodies [28]. However, necroptotic cells possessed the features of necrosis including loss of plasma membrane integrity, and intact nuclear membrane, electron-lucent cytoplasm [6]. In this study, we found that the characteristics of shinkonin treated glioma C6 cells were consistent with above description about necrosis. Double staining with Hoechst 33342 and PI is often used to distinguish necrosis from apoptosis [26]. Thus, the morphological findings in this study supported as well that shikonin induced necroptosis in C6 glioma cells.

Shikonin has been demonstrated to induce apoptosis in various tumor cell lines, until Han et al reported that shikonin could induce necroptosis in human leukemia cell lines [14]. Although Chen et al found shikonin induced apoptosis in human glioma cell lines, they did not examine whether necroptosis was induced as well in glioma cells [18]. Flow cytometry is an effective method to quantitatively distinguish cellular groups between necrosis and apoptosis. The data in this study acquired by flow cytometry showed that necrotic cells were almost totally prohibited by incubation with Nec-1 before exposure to shikonin. Additionally, pretreatment with Nec-1 made the C6 glioma cells at the stage of late apoptosis reduce from 26.02% to 13.60% at 3 µmol/L shikonin and from 37.32% to 24.17% at 6 µmol/L shikonin; made U87 cells at the stage of late apoptosis decrease from 33.37% to 7.96% at 5 µmol/L shikonin and from 42.90% to 27.14% at 10 µmol/L shikonin. As a potent necroptosis inhibitor, Nec-1 was previously thought not to inhibit apoptosis and could be used to differentiate necroptosis from apoptosis [8], despite that Wang YQ et al found that Nec-1 suppressed apoptosis in mice traumatic brain injury model [29]. Moreover, our results (Figure 3B) showed that Nec-1 did not inhibit apoptosis. By contrast, it was reported that shikonin induced necroptosis was partially switched to apoptosis by Nec-1 in leukemia cells [14], suggesting that Nec-1 did not inhibit apoptosis caused by shikonin. Thus, these evidences indicate that the glioma cells at the stage of late apoptosis detected by flow cytometry should be necroptotic cells. Therefore, we think that necroptosis is the major form of glioma cells death caused by shikonin at either lower or higher concentration in a short time.

Despite we did not investigate further the mechanism responsible for shikonin-induced apoptosis in C6 glioma cells, but we found that pretreatment with caspase inhibitor z-VAD-fmk did not alter the cellular numbers at the stage of late apoptosis, indicating the apoptosis caused by shikonin was not caspase-dependent. However, Chen et al found that the expression of PARP was up-regulated by shikonin in human glioma cell lines [18]. Mao et al demonstrated that JNK pathway was involved the apoptosis caused by shikonin in leukemia cells [30]. Thus, shikonin induced apoptosis in C6 glioma cells might be via multiple pathways.

Although the underlying mechanisms of necroptosis remain poorly understood, it is thought that necroptosis has its own unique signaling pathway which required the involvement of receptor interaction protein kinases 1 and 3 (RIP1 and RIP3), and can be specifically inhibited by necrostatins [6], [31]. Previous studies did not demonstrate the pattern of the expression of receptor interaction protein kinases caused by shikonin [14], but our results in this study showed that the expression of RIP-1 was up-regulated by shikonin in a dose and time dependent manner. Particularly, both the cell death and the expressional level of RIP-1 were attenuated by Nec-1. These results indicate that shikonin induced cell death in glioma cells is via activation of RIP-1. Moreover, other reports have also shown that necroptosis could be induced via modulating RIP-1 alone [32]–[34]. Nehs et al reported that radiation induced necroptosis in normal anaplastic thyroid and adrenocortical cancer cell lines but not in H295R cells deficient in receptor interacting protein 1 (RIP1) [33]. Saddoughi et al demonstrated that sphingosine analogue drug FTY720 induced necroptosis in lung cancer cells, which was inhibited by necrostatin-1, and knockdown or genetic loss of RIP-1 [34]. Despite we did not examine the effects of shikonin on the expression of RIP-3, it has been proved by some studies to be involved in necroptosis as well. Murine cytomegalovirus infection was demonstrated to induce necroptosis in infected cells in a RIP-3 dependent, RIP-1 independent manner [35]. Cyclosporin A induced necroptosis in NRK-52E cells was also via up-regulating the expression of RIP-3 [13].

Although either RIP-1 or RIP-3 could modulate necroptosis, their downstream signals are still unclear [6], [23]. In this study, we found that the ROS level induced by shikonin was not only dependent on shikonin concentration, but also in accordance with the expressional level of RIP-1. By contrast, inhibition of RIP-1 by Nec-1 suppressed the production of ROS caused by shikonin. Moreover, flow cytometry data showed that shikonin induced necroptosis in glioma cells was attenuated by anti-oxidant NAC at the concentration of inhibiting oxidative stress. Particularly, we found that anti-oxidant NAC could modulate the expression of RIP-1, indicating ROS is not only the targets modulated by RIP-1, but also could enhance the expression of RIP-1, which might form a loop leading to necroptosis. Therefore, we think that oxidative stress is involved in the shikonin induced necroptosis in glioma cells. Similar to our finding, Kim SK et al reported that oxidative stress participated palmitate induced RIP1-dependent necrosis [24]. Kim S et al thought that RIP-1 kinase activation is an upstream event of oxidative stress, because they found that RIP1 kinase could mediate arachidonic acid-induced oxidative death of oligodendrocyte precursors [36]. A recent report showed that Nec-1 protected cells from necrosis by blocking the reduction of mitochondrial membrane potential (MMP) [37], which indirectly proved that RIP-1 could mediate oxidative stress. Therefore, on the basis of our data and previous reports, we think that oxidative stress participates in the shikonin-induced necroptosis.

## Conclusion

In this study, we demonstrated that the cell death caused by shikonin in glioma cells was mainly via induction of necroptosis. Moreover, not only RIP-1 pathway, but also oxidative stress participated in the activation of necroptosis induced by shikonin. Thus, shikonin is a potential medicine that could be used in the future treatment of glioma.
