# Expression of Concern: Shikonin Kills Glioma Cells through Necroptosis Mediated by RIP-1

**DOI:** 10.1371/journal.pone.0343458

**Published:** 2026-02-23

**Authors:** The PLOS One Editors

Following the publication of this article [1], concerns were raised with results presented in Fig 6. Specifically,

- Fig 6A β-actin lanes 2-4 appear similar to Fig 6D β-actin lanes 3-5.
- β-actin lanes 1–4 in Fig 6H in [1] appear similar to PARP lanes 2–5 in Fig 4A in [2].

In response to the above concerns, the corresponding author stated that the wrong images were used. They provided an updated version of Fig 6 using data from later repeat experiments (S1 File) and what is stated to be original image files underlying the blots in the updated Fig 6 (S2 File).

In the absence of original uncropped and unadjusted image files captured at the time of the original experiments for all panels in the updated Fig 6, and individual-level quantitative data from which all graphs in the updated Fig 6 were generated, the above concerns remain unresolved and the *PLOS One* Editors issue this Expression of Concern.

## Supporting information

S1 File An updated version of Fig 6 with data from later repeat experiments. (TIF)

S2 File Cropped blots in support of the updated Fig 6 in S1 File. (ZIP)
