# Supplementary figures and images for: Expression of Concern: Shikonin Kills Glioma Cells through Necroptosis Mediated by RIP-1
Source: PLoS One. 2026 Feb 23;21(2):e0343458. doi: 10.1371/journal.pone.0343458 (PMC12928433; doi:10.1371/journal.pone.0343458)

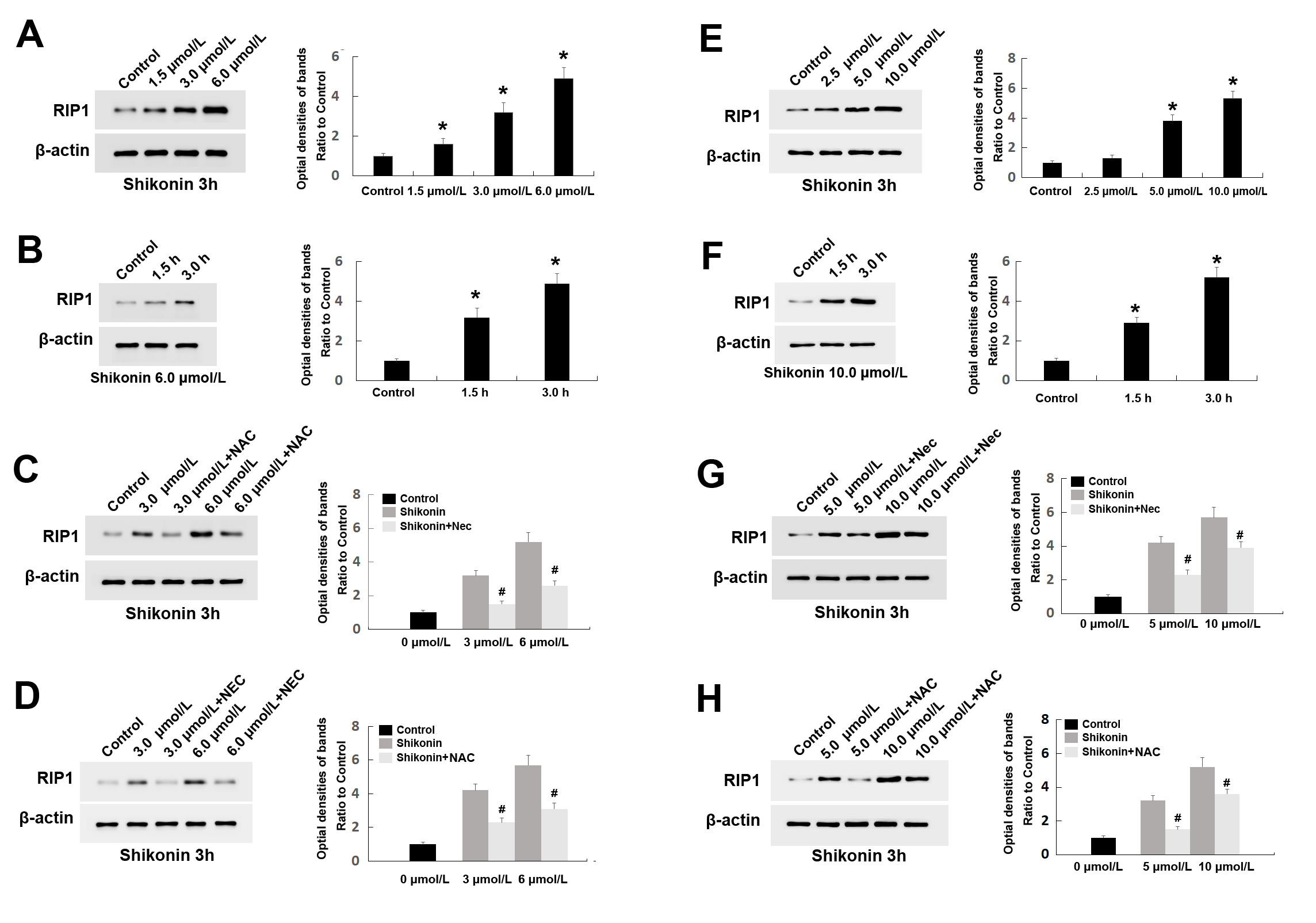

Supplement: S1 File — (TIF) [file pone.0343458.s001.tif]

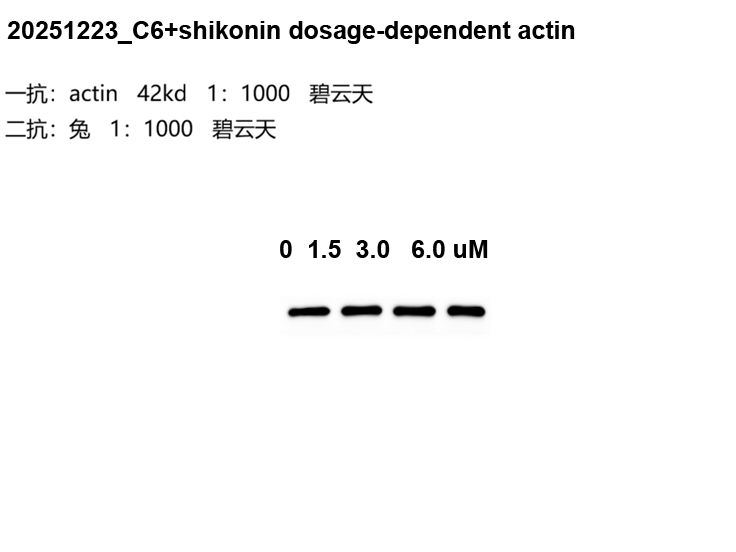

Supplement: S2 File — (ZIP) [file pone.0343458.s002.zip › FIgure 6A_C6+shikonin dosage-dependent actin.tif]

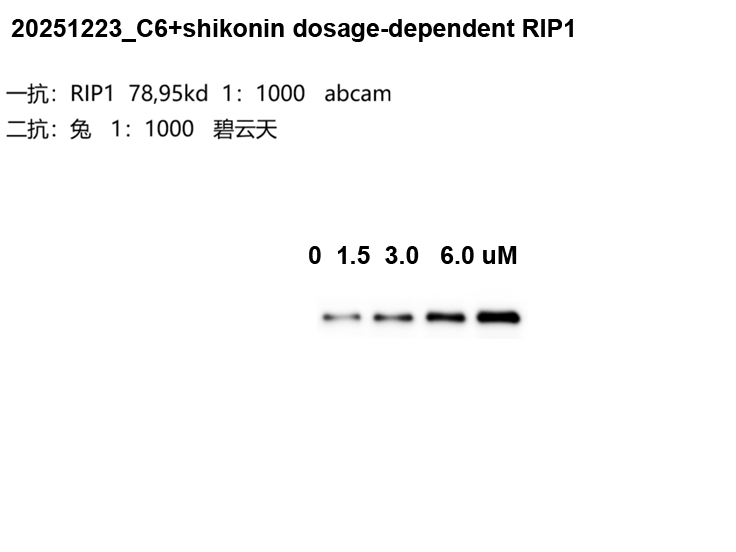

Supplement: S2 File — (ZIP) [file pone.0343458.s002.zip › FIgure 6A_C6+shikonin dosage-dependent RIP1.tif]

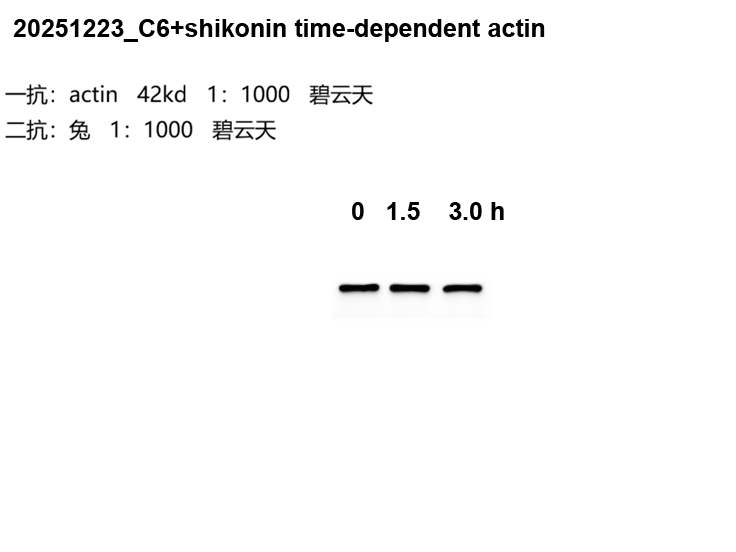

Supplement: S2 File — (ZIP) [file pone.0343458.s002.zip › FIgure 6B_C6+shikonin time-dependent actin.tif]

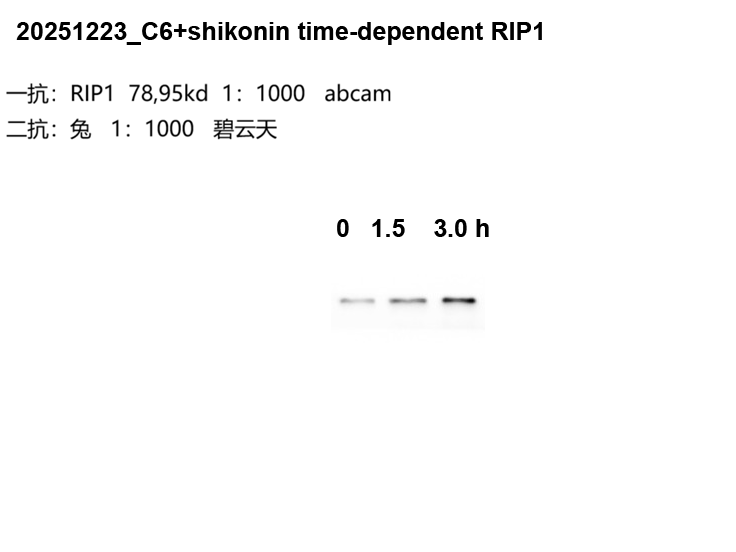

Supplement: S2 File — (ZIP) [file pone.0343458.s002.zip › FIgure 6B_C6+shikonin time-dependent RIP1.tif]

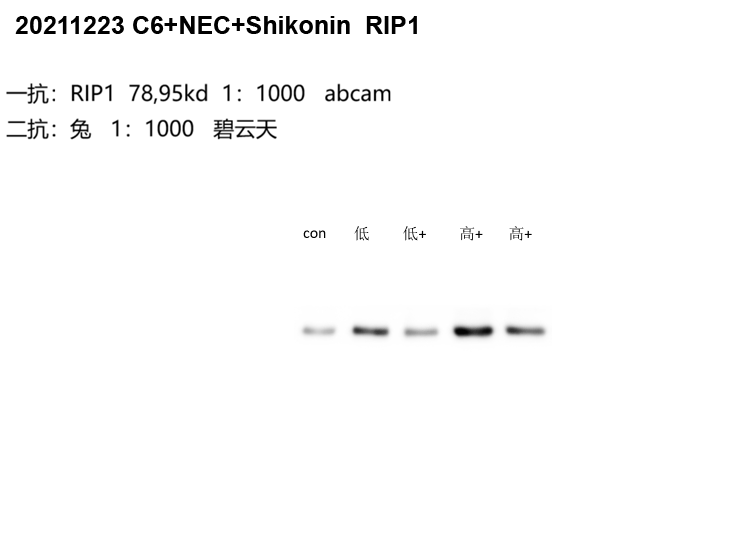

Supplement: S2 File — (ZIP) [file pone.0343458.s002.zip › FIgure 6C C6+NEC+shikonin RIP1.tif]

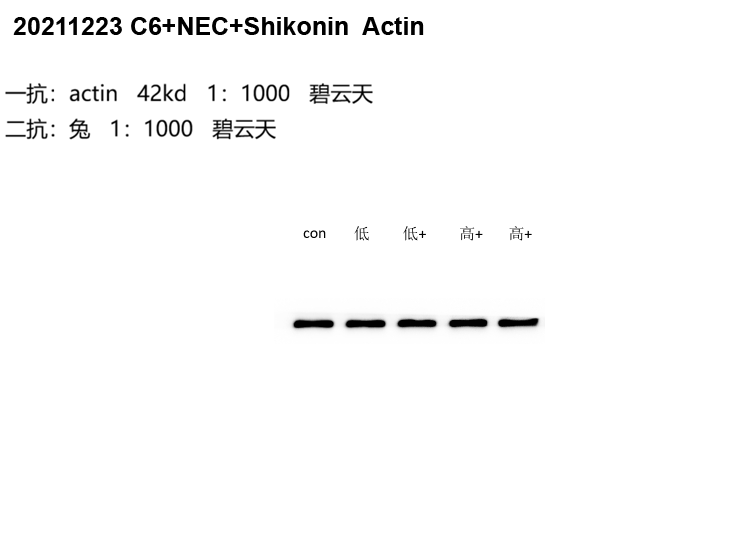

Supplement: S2 File — (ZIP) [file pone.0343458.s002.zip › FIgure 6C_C6+NEC+shikonin actin.tif]

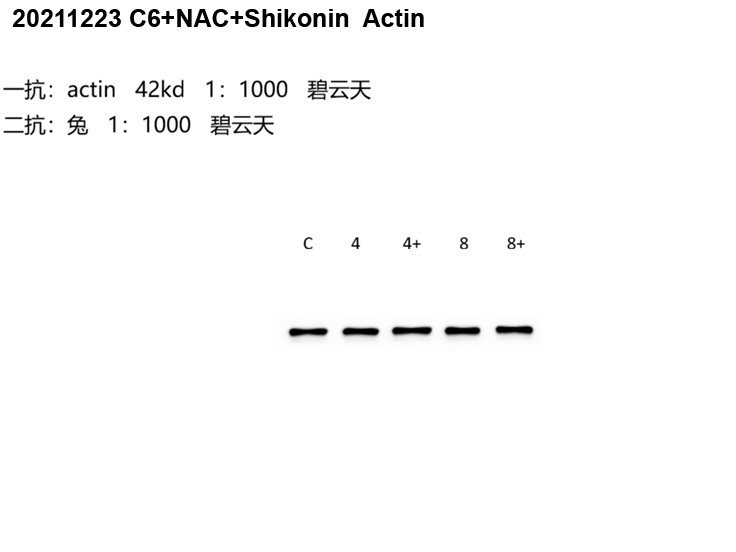

Supplement: S2 File — (ZIP) [file pone.0343458.s002.zip › FIgure 6D _C6+NAC+shikonin actin.tif]

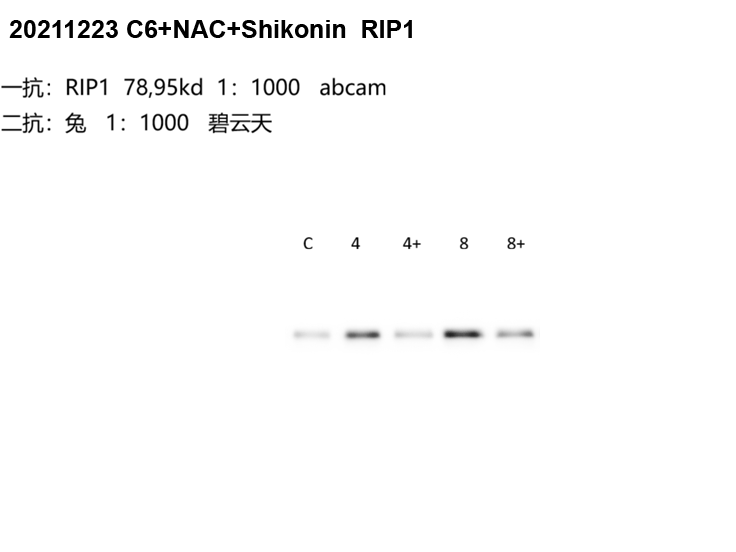

Supplement: S2 File — (ZIP) [file pone.0343458.s002.zip › FIgure 6D_C6+NAC+shikonin RIP1.tif]

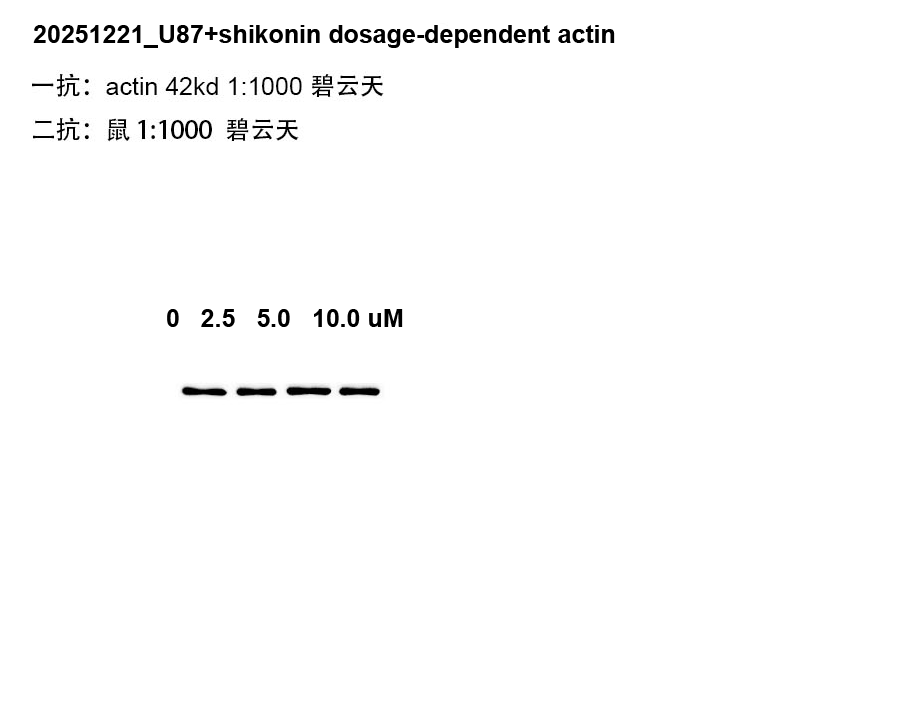

Supplement: S2 File — (ZIP) [file pone.0343458.s002.zip › FIgure 6E_U87+shikonin dosage-dependent actin.tif]

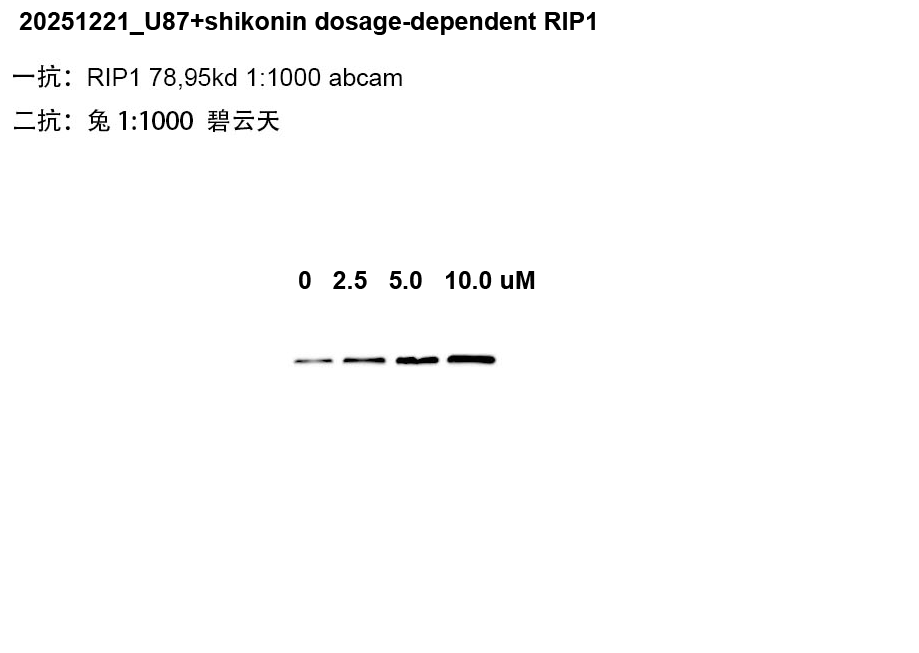

Supplement: S2 File — (ZIP) [file pone.0343458.s002.zip › FIgure 6E_U87+shikonin dosage-dependent RIP1.tif]

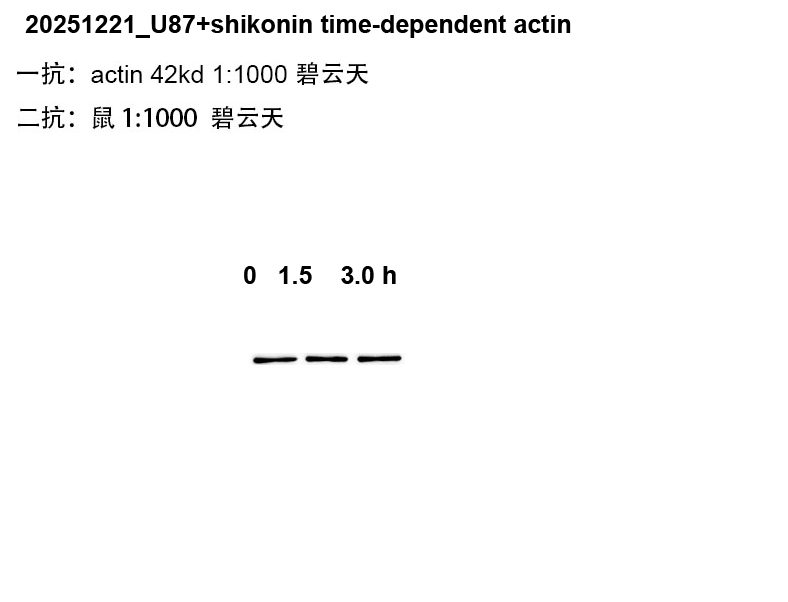

Supplement: S2 File — (ZIP) [file pone.0343458.s002.zip › FIgure 6F_U87+shikonin time-dependent actin.tif]

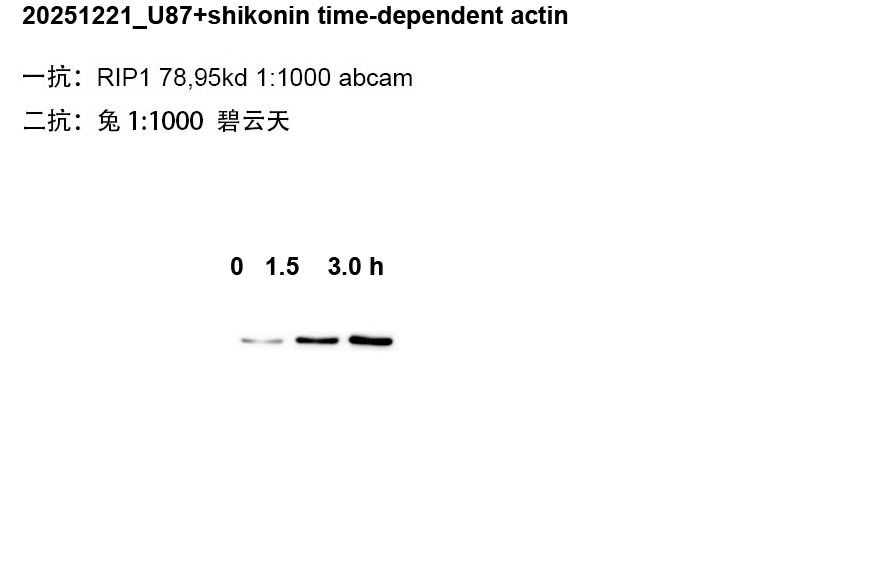

Supplement: S2 File — (ZIP) [file pone.0343458.s002.zip › FIgure 6F_U87+shikonin time-dependent RIP1.tif]

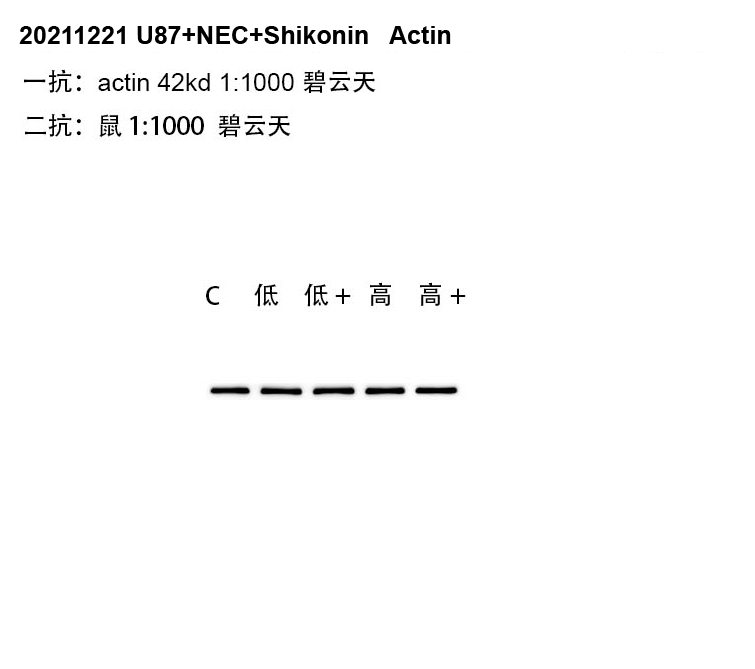

Supplement: S2 File — (ZIP) [file pone.0343458.s002.zip › FIgure 6G_U87+NEC+shikonin actin.tif]

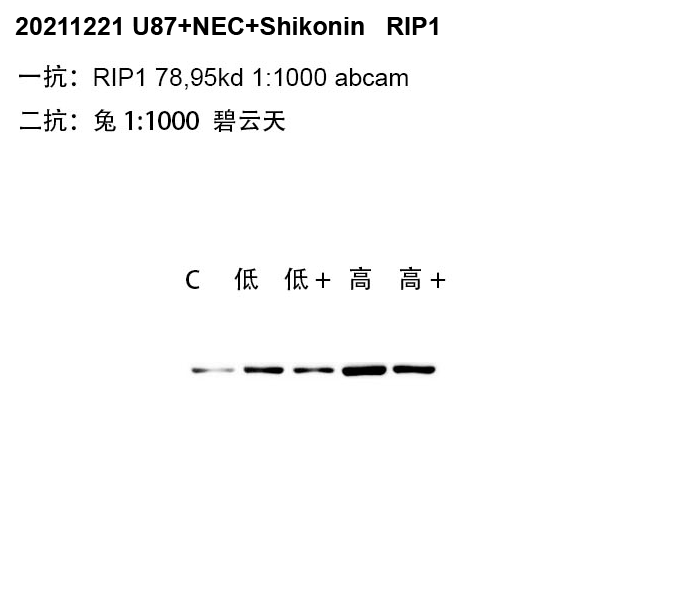

Supplement: S2 File — (ZIP) [file pone.0343458.s002.zip › FIgure 6G_U87+NEC+shikonin RIP1.tif]

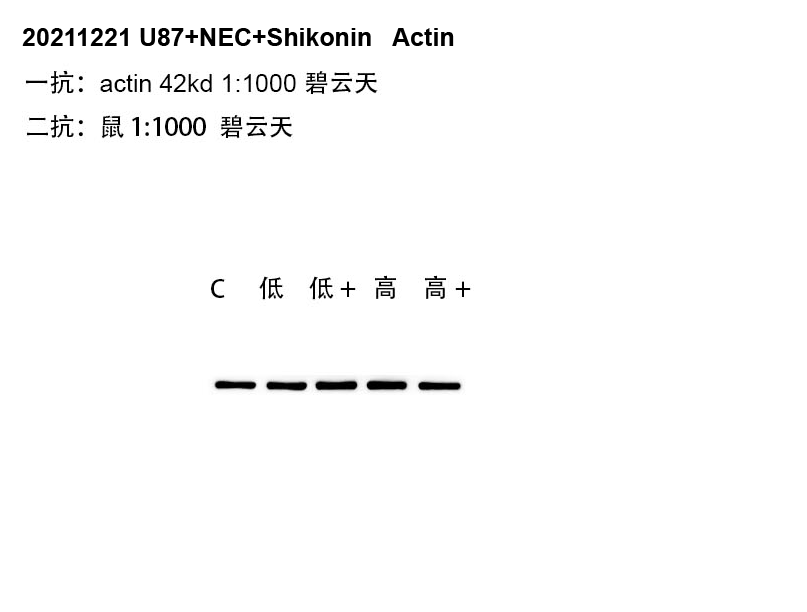

Supplement: S2 File — (ZIP) [file pone.0343458.s002.zip › FIgure 6H_U87+NAC+shikonin actin.tif]

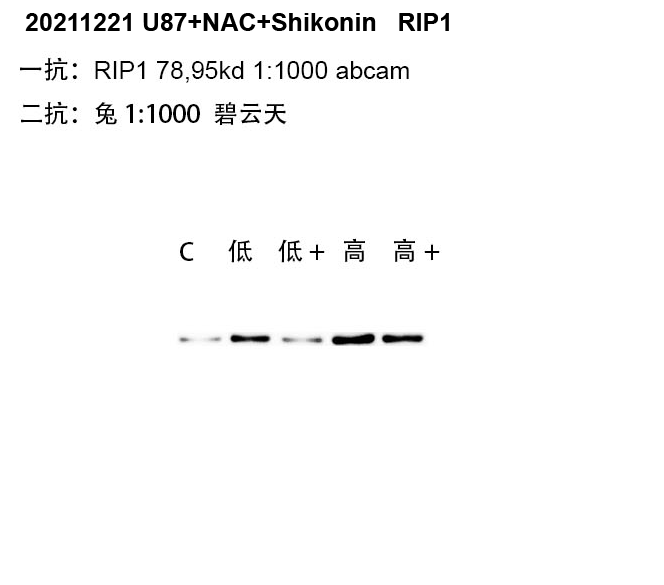

Supplement: S2 File — (ZIP) [file pone.0343458.s002.zip › FIgure 6H_U87+NAC+shikonin RIP1.tif]
